# Supplementary material for: A CRISPRi/a platform in human iPSC-derived microglia uncovers regulators of disease states
Source: Nat Neurosci. 2022 Aug 11;25(9):1149–62. doi: 10.1038/s41593-022-01131-4 (PMC9448678; doi:10.1038/s41593-022-01131-4)
Supplement: Supplementary file 1 — Supplementary Fig. 1 and discussion. [file 41593_2022_1131_MOESM1_ESM.pdf]

nature portfolio

https://doi.org/10.1038/s41593-022-01131-4

Supplementary information

A CRISPRi/a platform in human iPSC-derived microglia uncovers regulators of disease states

In the format provided by the authors and unedited

SUPPLEMENTARY INFORMATION

A CRISPRi/a platform in human iPSC-derived microglia uncovers regulators of disease states

Nina M. Dräger, Sydney M. Sattler, Cindy Tzu-Ling Huang, Olivia M. Teter, Kun Leng, Sayed Hadi Hashemi, Jason Hong, Giovanni Aviles, Claire D. Clelland, Lihong Zhan, Joe C. Udeochu, Lay Kodama, Andrew B. Singleton, Mike A. Nalls, Justin Ichida, Michael E. Ward, Faraz Faghri, Li Gan, Martin Kampmann

- **Supplementary Figure 1**
- **Supplementary Discussion**

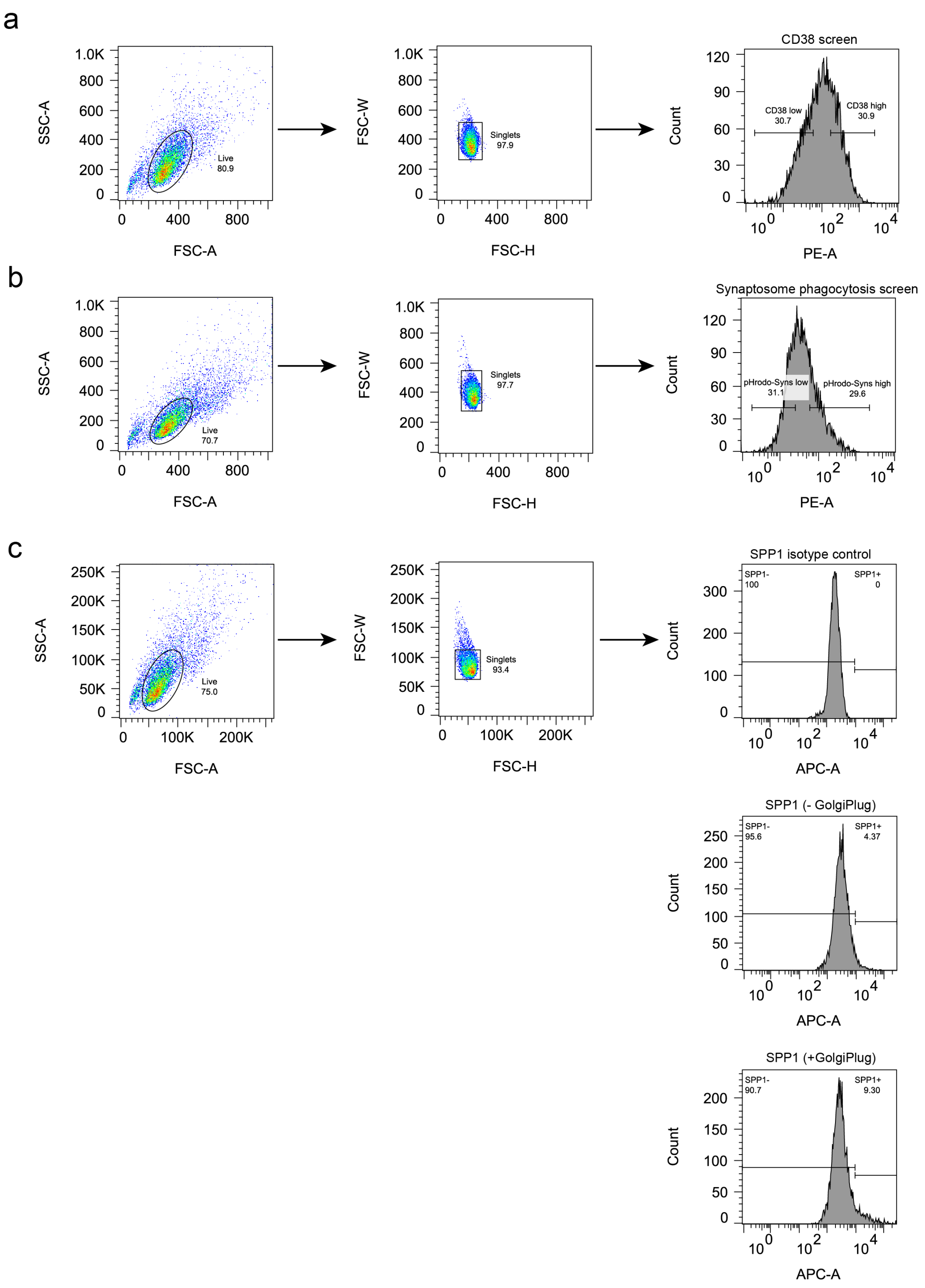

Supplementary Figure 1: Gating strategies for FACS-based screens and SPP1 positive cells. **a,b** Gating strategies for (a) CD38 screens and (b) synaptosome phagocytosis screens. Intact iTF-Microglia were identified from FSC-SSC plot and then gated for singlets. These cells were sorted into high and low signal populations corresponding to the top 30% and the bottom 30% of the signal distribution. **c,** To determine the fraction of SPP1+ cells, cells were treated with GolgiPlug and singlets were classified using the SPP1 isotype control to determine the threshold.

SUPPLEMENTARY DISCUSSION

Areas for methodological improvement

Improved inducible CRISPRi/a machinery with more potent gene repression and activation in fully differentiated iTF-Microglia would enable the induction of CRISPRi/a at later stages during differentiation to avoid false-positive hits that affect microglial differentiation, such as *CDK8* and *TGFBR2* (Fig. 4b, Extended Data Fig. 5b-f).

Another goal for future technology development is further acceleration and enhancement of the microglial maturation. One potential concern about sustained expression of transgenic transcription factors is that this could promote certain microglial states over others. A protocol in which transcription factor expression is discontinued after day 8 (Extended Data Fig. 1a) can mitigate this concern. As with all currently available *in vitro* culture systems, microglia are slightly activated in monoculture and lose their unique homeostatic brain signature[1]. Previous research has shown that iPSC-microglia become more homeostatic in co-culture with neurons[2], which is compatible with our own observation of enhanced ramification of iTF-Microglia in neuronal co-culture (Fig. 2f). Alternatively, optimizing the set of transcription factors used to generate iTF-Microglia may result in improved abundance of homeostatic microglia. CRISPRa screens in our current platform are a scalable strategy to identify additional transcription factors to promote microglial maturation and homeostasis, leading to ever more faithful models of human microglia.

Phagocytosis phenotypes of disease-associated genes

Coding mutations in profilin 1 (*PFN1*) gene cause amyotrophic lateral sclerosis (ALS)[3]. *PFN1* is a small actin-binding protein that promotes formin-based actin polymerization and regulates numerous cellular functions, but how mutations in *PFN1* cause ALS is unclear. The actin cytoskeleton is known to be important for the physiological functions of microglia, including migration and phagocytosis. We observed that *PFN1* overexpression disrupts the actin cytoskeleton in iTF-Microglia with higher levels of F-actin. Recently, a study has shown that *PFN1* is also involved in microglia activation, since knockdown of *PFN1* inhibited M1 proinflammatory microglial polarization and promoted anti-inflammatory M2 microglia polarization after oxygen and glucose deprivation[4]. Introducing the ALS-associated mutations in the *PFN1* gene in iPSCs will shed light on the impact of these specific mutations on the function of different relevant cell types, such as iPSC-derived neurons and microglia.

Genetic variants in the *INPP5D* locus are associated with an increased susceptibility to AD[5] and cerebrovascular function as well as tau and Aβ levels in the cerebrospinal fluid of AD patients[6]. *INPP5D* encodes the lipid phosphatase SHIP1, which is selectively expressed in brain microglia. SHIP1 inhibits signal transduction initiated by activation of immune cell surface receptors, such as TREM2[7]. Intriguingly, *INPP5D* expression increases with AD progression, predominantly in plaque-associated microglia, and correlates with plaque density[8]. Given the results from our phagocytosis screen, *INPP5D* overexpression might result in microglia with deficient phagocytic capacity, resulting in increased Aβ deposition and neurodegeneration. Concordant with the

findings from our genetic screen, a recent study found that pharmacological SHIP1/2 inhibitors promote microglial phagocytosis *in vitro* and *in vivo*[9].

Regulators of the SPP1 state

Knockdown or pharmacological inhibition of *MAPK14* strongly promoted adoption of the disease-associated SPP1-positive state. Previous work suggested a functional connection between SPP1 and MAPK14 in cancer cells, where SPP1 can activate the p38 MAPK signaling pathway, which comprises MAPK14[10]. MAPK14 was also recently predicted to be a unique network regulator in DAM[11]. However, our identification of MAPK14 as a regulator of the SPP1+ state is novel and enhances our understanding of modulators of microglia cell states.

We found that the SPP1-positive microglia state can be selectively depleted by genetic and pharmacological inhibition of CSF1R. CSF1R inhibitors have beneficial effects in mouse models of diseases including AD[12, 13], tauopathy[14] and MS[15]. Intriguingly, CSF1R inhibition reduced SPP1 expression in the MS model, while homeostatic genes such as *TMEM119* and *P2RY12* were increased[15], paralleling our finding that the SPP1 microglia state is selectively vulnerable to CSF1R inhibition. Additionally, disruption of CSF1-CSF1R signaling downregulated SPP1 in the cerebellum[16]. Combining CSF1R depletion and single cell profiling has enabled us previously to elucidate the differential effects of CSF1R inhibitors on microglia subtypes[17]. Following CSF1R inhibition, we found an enrichment of microglia states with elevated markers of inflammatory chemokines and proliferation and interestingly, in concordance with our findings in iTF-Microglia here, an upregulation of cell surface receptor CD74[17]. Others have reported compensatory upregulation of TREM2/b-catenin and IL-34 in microglia following conditional CSF1R KO[18]; however, we did not find consistent upregulation of these factors in our iTF-microglia (Supplementary Table 9). Based on our new finding that CSF1R inhibition at low doses that are nontoxic to most microglia selectively depletes the SPP1+ population in iTF-Microglia, low-dose CSF1R inhibition might also give us a tool to study the SPP1+ population in mouse disease models.

References

1. Bohlen, C.J., *et al.* Diverse Requirements for Microglial Survival, Specification, and Function Revealed by Defined-Medium Cultures. *Neuron* **94**, 759-773.e758 (2017).

2. Haenseler, W., *et al.* A Highly Efficient Human Pluripotent Stem Cell Microglia Model Displays a Neuronal-Co-culture-Specific Expression Profile and Inflammatory Response. *Stem Cell Reports* **8**, 1727-1742 (2017).

3. Wu, C.H., *et al.* Mutations in the profilin 1 gene cause familial amyotrophic lateral sclerosis. *Nature* **488**, 499-503 (2012).

4. Lu, E., *et al.* Profilin 1 knockdown prevents ischemic brain damage by promoting M2 microglial polarization associated with the RhoA/ROCK pathway. *J Neurosci Res* **98**, 1198-1212 (2020).

5. Lambert, J.C., *et al.* Meta-analysis of 74,046 individuals identifies 11 new susceptibility loci for Alzheimer's disease. *Nat Genet* **45**, 1452-1458 (2013).

6. Yao, X., *et al.* Targeted genetic analysis of cerebral blood flow imaging phenotypes implicates the INPP5D gene. *Neurobiol Aging* **81**, 213-221 (2019).

7. Peng, Q., *et al.* TREM2- and DAP12-dependent activation of PI3K requires DAP10 and is inhibited by SHIP1. *Sci Signal* **3**, ra38 (2010).

8. Tsai, A.P., *et al.* INPP5D expression is associated with risk for Alzheimer's disease and induced by plaque-associated microglia. *Neurobiol Dis* **153**, 105303 (2021).

9. Pedicone, C., *et al.* Pan-SHIP1/2 inhibitors promote microglia effector functions essential for CNS homeostasis. *J Cell Sci* **133** (2020).

10. Huang, R.H., *et al.* Osteopontin Promotes Cell Migration and Invasion, and Inhibits Apoptosis and Autophagy in Colorectal Cancer by activating the p38 MAPK Signaling Pathway. *Cell Physiol Biochem* **41**, 1851-1864 (2017).

11. Xu, J., *et al.* Multimodal single-cell/nucleus RNA sequencing data analysis uncovers molecular networks between disease-associated microglia and astrocytes with implications for drug repurposing in Alzheimer's disease. *Genome Res* (2021).

12. Spangenberg, E., *et al.* Sustained microglial depletion with CSF1R inhibitor impairs parenchymal plaque development in an Alzheimer's disease model. *Nat Commun* **10**, 3758 (2019).

13. Sosna, J., *et al.* Early long-term administration of the CSF1R inhibitor PLX3397 ablates microglia and reduces accumulation of intraneuronal amyloid, neuritic plaque deposition and pre-fibrillar oligomers in 5XFAD mouse model of Alzheimer's disease. *Mol Neurodegener* **13**, 11 (2018).

14. Johnson, N.R., *et al.* Sex-specific life extension in tauopathy mice by CSF1R inhibition causing selective microglial depletion and suppressed pathogenesis. *bioRxiv*, 2021.2003.2020.436288 (2021).

15. Hagan, N., *et al.* CSF1R signaling is a regulator of pathogenesis in progressive MS. *Cell Death Dis* **11**, 904 (2020).

16. Kana, V., *et al.* CSF-1 controls cerebellar microglia and is required for motor function and social interaction. *J Exp Med* **216**, 2265-2281 (2019).

17. Zhan, L., *et al.* A MAC2-positive progenitor-like microglial population is resistant to CSF1R inhibition in adult mouse brain. *Elife* **9** (2020).

18. Pons, V., Lévesque, P., Plante, M.M. & Rivest, S. Conditional genetic deletion of CSF1 receptor in microglia ameliorates the physiopathology of Alzheimer's disease. *Alzheimers Res Ther* **13**, 8 (2021).
