# Supplementary material for: A CRISPRi/a platform in human iPSC-derived microglia uncovers regulators of disease states
Source: Nat Neurosci. 2022 Aug 11;25(9):1149–62. doi: 10.1038/s41593-022-01131-4 (PMC9448678; doi:10.1038/s41593-022-01131-4)
Supplement: Supplementary file 2 — Reporting Summary [file 41593_2022_1131_MOESM2_ESM.pdf]

nature research

Corresponding author(s): Martin Kampmann, Li Gan

Last updated by author(s): Jun 23, 2022

Reporting Summary

Nature Research wishes to improve the reproducibility of the work that we publish. This form provides structure for consistency and transparency in reporting. For further information on Nature Research policies, see Authors & Referees and the Editorial Policy Checklist.

Statistics

For all statistical analyses, confirm that the following items are present in the figure legend, table legend, main text, or Methods section.

n/a	Confirmed	
☐	☒	The exact sample size (n) for each experimental group/condition, given as a discrete number and unit of measurement
☐	☒	A statement on whether measurements were taken from distinct samples or whether the same sample was measured repeatedly
☐	☒	The statistical test(s) used AND whether they are one- or two-sided<br>*Only common tests should be described solely by name; describe more complex techniques in the Methods section.*
☒	☐	A description of all covariates tested
☐	☒	A description of any assumptions or corrections, such as tests of normality and adjustment for multiple comparisons
☐	☒	A full description of the statistical parameters including central tendency (e.g. means) or other basic estimates (e.g. regression coefficient) AND variation (e.g. standard deviation) or associated estimates of uncertainty (e.g. confidence intervals)
☐	☒	For null hypothesis testing, the test statistic (e.g. F, t, r) with confidence intervals, effect sizes, degrees of freedom and P value noted<br>*Give P values as exact values whenever suitable.*
☒	☐	For Bayesian analysis, information on the choice of priors and Markov chain Monte Carlo settings
☒	☐	For hierarchical and complex designs, identification of the appropriate level for tests and full reporting of outcomes
☐	☒	Estimates of effect sizes (e.g. Cohen's d, Pearson's r), indicating how they were calculated

Our web collection on statistics for biologists contains articles on many of the points above.

Software and code

Policy information about availability of computer code

Data collection	For FACS data collection, we used BD FACSDiva (version 8.0.1.1). For microscopy data collection by InCell 6000, we used In Cell Analyzer 6000 Acquisition Software (version 4.0). For qPCR data collection, we used Quant Studio Real Time PCR software (version 1.3). For cytokine array imaging, we used Image Studio (version 5.2). For Cell-TiterGlo luminescence data collection, we used SoftMax Pro 6.5.1.
Data analysis	For FACS analysis, we used FlowJo (version 10.7.1) and Prism 8 (8.4.2). For microscopy image analysis we used Fiji (version 2.0.0) and CellProfiler (version 4.1.3). For pooled CRISPR screen analysis, we used MAGeck-iNC (version 5.2) and Bowtie (version 0.12.9). For bulk RNA-seq analysis, we used Salmon (version 1.4.0), Tximport (version 1.18.0), ggplot2 (version 3.3.3), Complex Heatmap (version 2.6.2), DESeq2 (version 1.30.1), and VennDiagram (version 1.6.20). For CROP-seq analysis, we used Cell Ranger (version 5.0.1), in R (version 4.0.3) we used DropletUtils (version 1.10.3) and Seurat (4.0.1).<br>The MAGeCK-iNC bioinformatics pipeline for analysis of pooled screens available at https://kampmannlab.ucsf.edu/mageck-inc. The R notebooks for the RNA-seq and CROP-seq analysis are available at https://kampmannlab.ucsf.edu/article/microglia-analysis. The CellProfiler pipelines will be made available on request to the corresponding authors (MK), and will also be submitted to the CellProfiler depository of published pipelines (https://cellprofiler.org/examples/published_pipelines.html) upon publication.

For manuscripts utilizing custom algorithms or software that are central to the research but not yet described in published literature, software must be made available to editors/reviewers. We strongly encourage code deposition in a community repository (e.g. GitHub). See the Nature Research guidelines for submitting code & software for further information.

Data

Policy information about availability of data

All manuscripts must include a data availability statement. This statement should provide the following information, where applicable:

- Accession codes, unique identifiers, or web links for publicly available datasets
- A list of figures that have associated raw data
- A description of any restrictions on data availability

All screen datasets and RNA-transcriptomic datasets are publicly available in the CRISPRbrain data commons (http://crisprbrain.org/) (associated with Figures 1, 2, 4,

5, 6, 7 and Extended Data Figures 1, 4, 5, 6, 7). RNA sequencing datasets reported in this paper are available on NCBI GEO, accession number GSE178317 (https://www.ncbi.nlm.nih.gov/geo/query/acc.cgi?acc=GSE178317). There are no restrictions on data availability.

Field-specific reporting

Please select the one below that is the best fit for your research. If you are not sure, read the appropriate sections before making your selection.

☒ Life sciences ☐ Behavioural & social sciences ☐ Ecological, evolutionary & environmental sciences

For a reference copy of the document with all sections, see nature.com/documents/nr-reporting-summary-flat.pdf

Life sciences study design

All studies must disclose on these points even when the disclosure is negative.

Sample size	Sample sizes were determined by existing studies in the field to enable statistical analyses and reproducibility, specifically our previous CRISPRi screens in iPSC-derived neurons (Tian et al. 2019, Neuron 104:239-255).
Data exclusions	No data was excluded from the relevant analyses.
Replication	Major findings were validated using independent samples and orthogonal approaches. Numbers of replicates are listed in each Figure.
Randomization	No randomization of samples was used since treatment group of cells were generally derived from the same population of cells.
Blinding	Blinding was not relevant to our study since no subjective rating of data was involved. Instead, we used computational quantification.

Reporting for specific materials, systems and methods

We require information from authors about some types of materials, experimental systems and methods used in many studies. Here, indicate whether each material, system or method listed is relevant to your study. If you are not sure if a list item applies to your research, read the appropriate section before selecting a response.

Materials & experimental systems

n/a	Involved in the study	
☐	☒	Antibodies
☐	☒	Eukaryotic cell lines
☒	☐	Palaeontology
☒	☐	Animals and other organisms
☒	☐	Human research participants
☒	☐	Clinical data

Methods

n/a	Involved in the study	
☒	☐	ChIP-seq
☐	☒	Flow cytometry
☒	☐	MRI-based neuroimaging

Antibodies

Antibodies used

Primary antibodies: IBA-1 (1:1000, Wako; Cat. No. 019-19741), GPR34 (1:150, R&D Systems; Cat. No. MAB4617), TFRC (1:200, abcam ; Cat. No. ab84036), Synaptophysin (1:1000, Synaptic Systems; Cat. No. 101004).

Secondary antibodies: Alexa Fluor 488 goat anti rabbit IgG (1:500, Invitrogen; Cat. No. A11034), Alexa Fluor 488 goat anti mouse IgG (1:500, Invitrogen; Cat. No. A11029), Alexa Fluor 568 goat anti rabbit IgG (1:500, Invitrogen; Cat. No. A11036), Alexa Fluor 647 goat anti-chicken IgG (1:500 dilution; abcam Cat. No. ab 150171)

Coupled antibodies: PE-Cy7 anti-human CD71 (TFRC) (1: 66, BioLegend; Cat. No. 334112), PE/Cy7 anti-human CD184 (CXCR4) (1:66, BioLegend; Cat. No. 306514), Human CCL13 488 (1:75, R&D Systems; Cat. No. IC327G), hCD38 PE (1:200, R&D Systems; Cat. No. FAB2404P), hOsteopontin eFluor 660 (1:75, eBioscience ; Cat. No. 50-9096-42).

Validation

Antibodies were validated by the manufacturers as follows: Anti-IBA-1 (Wako; Cat. No. 019-19741) was validated by immunohistochemistry of microglia in rat cerebral cortex and mouse cerebellum. Anti-GPR34 (R&D Systems; Cat. No. MAB4617) has been validated by staining human GPR34 transfectants but not irrelevant transfectants. Anti-Synaptophysin (Synaptic Systems; Cat. No. 101004) was validated by western blotting of a synaptosomal fraction of rat brains and validated by immunostaining of hippocampal neurons. Anti-TFRC (abcam ; Cat. No. ab84036) was validated by western blotting of various cell lysates, detecting a clear band of approximately 98 kDa (predicted molecular weight: 84 kDa).

Eukaryotic cell lines

Policy information about cell lines

Cell line source(s)	The human iPSC line WTc11 was obtained from Coriell (GM25256). The human iPSC line WTc11 was obtained with CRISPRi machinery was obtained from Coriell (AICS-0090-391). HEK293T cells were obtained from ATCC (CRL-3216).
Authentication	iPSCs were karyotyped (Extended Data Fig. 3) and characterized for maintaining capability to differentiate into iTF-Microglia. AICS-0090 cl.391 was authenticated by the Allen Institute for Cell Science. We did not authenticate the HEK293T cells.
Mycoplasma contamination	All lines were tested negative for mycoplasma using the Universal Mycoplasma Detection Kit (ATCC 30-1012KTM).
Commonly misidentified lines (See ICLAC register)	No commonly misidentified lines were used.

Flow Cytometry

Plots

Confirm that:

☒ The axis labels state the marker and fluorochrome used (e.g. CD4-FITC).

☒ The axis scales are clearly visible. Include numbers along axes only for bottom left plot of group (a 'group' is an analysis of identical markers).

☒ All plots are contour plots with outliers or pseudocolor plots.

☒ A numerical value for number of cells or percentage (with statistics) is provided.

Methodology

Sample preparation	Cells were dissociated using Accutase (for iPSCs) or TryplE (for iTF-Microglia) and analyzed or sorted by flow cytometry based on staining signals. For cell surface receptor stainings, dissociated and in DPBS resuspended iTF-Microglia were blocked for 15 min with 1:20 Human FC Block (BD Biosciences; Cat. No. 564220). For CRISPRi/a validation, cells were stained with 1:66 PE/Cy7 anti-human CD184 (CXCR4) (BioLegend; Cat. No. 306514) or 1:66 PE-Cy7 anti-human CD71 (TFRC) (BioLegend; Cat. No. 334112) for 30 min in the dark. Cells were washed twice with DPBS before analyzing them by flow cytometry. For the CD38 screen and validation experiments, iTF-Microglia were stained with 1:200 anti-hCD38 PE (R&D Systems; Cat. No. FAB2404P). Cells were washed twice with DPBS before analyzing them by flow cytometry.<br>For intracellular protein staining, dissociated iTF-Microglia were fixed and permeabilized with the eBioscience Intracellular Fixation and Permeabilization Buffer Set (Invitrogen; Cat. No. 88-8824-00) according to the manufacturer's instructions. Cells were stained with 1:75 Anti-Hu Osteopontin (SPP1) eFluor 660 (eBioscience; Cat. No. 50-9096-42) or 1:75 Human CCL13 488 (R&D Systems; Cat. No. IC327G) or their isotype controls Mouse IgG1 Control Alexa Fluor 488 conjugated (R&D Systems; Cat. No. IC002G) and Mouse IGG1 kappa Isotype (eBioscience; Cat. No. 50-4714-82) over night at 4°C. Cells were washed twice with DPBS before analyzing them by flow cytometry. For analyzing phagocytosis rates and the phagocytosis screen, cells were washed twice with DPBS, dissociated, resuspended in ice-cold DPBS, and then analyzed via flow cytometry.
Instrument	BD LSRFortessa X14 and BD FACSAria Fusion
Software	BD FACSDiva (version 8.0.1.1) and FlowJo (version 10.4)
Cell population abundance	To analyze staining signals or uptake of fluorescent materials (beads or synaptosomes), cells were analyzed and not sorted. The starting populations were pure (either iPSCs or iTF-Microglia). For pooled screens, iTF-Microglia were sorted into high and low signal populations corresponding to the top 30% and the bottom 30% of the staining signal distribution.
Gating strategy	Preliminary FSC/SSC gates encompassed all live, single cells in the population. For pooled screens, these cells were sorted into high and low signal populations corresponding to the top 30% and the bottom 30% of the staining signal distribution.

☒ Tick this box to confirm that a figure exemplifying the gating strategy is provided in the Supplementary Information.
