# Supplementary material for: A CRISPRi/a platform in human iPSC-derived microglia uncovers regulators of disease states
Source: Nat Neurosci. 2022 Aug 11;25(9):1149–62. doi: 10.1038/s41593-022-01131-4 (PMC9448678; doi:10.1038/s41593-022-01131-4)
Supplement: Supplementary file 3 — Editorial Assessment Report [file 41593_2022_1131_MOESM3_ESM.pdf]

nature portfolio GUIDED OPEN ACCESS

Contents of this report

- **Manuscript details**: overview of your manuscript and the editorial team.
- **Review synthesis**: summary of the reviewer reports provided by the editors.
- **Editorial recommendation**: personalized evaluation and recommendation from all 3 journals.
- **Annotated reviewer comments**: the referee reports with comments from the editors.
- **Open research evaluation**: advice for adhering to best reproducibility practices.

About the editorial process

Because you selected the **Nature Portfolio Guided Open Access option**, your manuscript was assessed for suitability in three of our titles publishing high-quality work across your field of research. More information about Guided Open Access can be found **here**.

Collaborative editorial assessment

Your editorial team discussed the manuscript to determine its suitability for the Nature Portfolio Guided OA pilot. Our assessment of your manuscript takes into account several factors, including whether the work meets the **technical standard** of the Nature Portfolio and whether the findings are of **immediate significance** to the readership of at least one of the participating journals in the Guided OA pilot.

Peer review

Experts were asked to evaluate the following aspects of your manuscript:

- **Novelty** in comparison to prior publications;
- **Likely audience** of researchers in terms of broad fields of study and size;
- **Potential impact** of the study on the immediate or wider research field;
- **Evidence** for the claims and whether additional experiments or analyses could feasibly strengthen the evidence;
- **Methodological detail** and whether the manuscript is reproducible as written;
- Appropriateness of the literature review.

Editorial evaluation of reviews

Your editorial team discussed the potential suitability of your manuscript for each of the participating journals. They then discussed the revisions necessary in order for the work to be published, keeping each journal's specific editorial criteria in mind.

Journals in the Nature portfolio will support authors wishing to transfer their reviews and (where reviewers agree) the reviewers' identities to journals outside of Springer Nature.

If you have any questions about review portability, please contact our editorial office at guidedoa@nature.com.

Manuscript details

Tracking number	Submission date	Decision date
GUIDEDOA-21-00163	22 June 2021	Click or tap to enter a date.

**Title**	A CRISPRi/a platform in iPSC-derived microglia uncovers regulators of disease states	**Corresponding author**	Martin Kampmann<br>**Affiliation:** University of California, San Francisco
**Preprint information**	There is a preprint of this manuscript posted at https://www.biorxiv.org/content/10.1101/2021.06.16.448639v1.full]	**Peer review type**	Single-blind

Editorial assessment team

**Primary editor**	**Shari Wiseman**<br>**Home Journal:** *Nature Neuroscience*, ORCID: 0000-0001-7868-9723<br>**Email:** shari.wiseman@us.nature.com
**Editorial team members**	**David Rowland**, *Nature*, ORCID:<br>**Elisa Floriddia**, *Nature Communications*, ORCID: 0000-0003-2304-8114
**About your primary editor**	Shari Wiseman received her PhD from Yale University, where she worked with Dr. Angus Nairn. She employed biochemical, proteomic, and behavioral approaches to examine signal transduction mechanisms that regulate neuronal protein synthesis. She then went on to postdoctoral research at Beth Israel-Deaconess Medical Center/Harvard Medical School investigating animal models of autism spectrum disorders, followed by additional postdoctoral training in Dr. Stephen Moss's lab at Tufts University, where she studied the regulation of GABAB receptors by excitotoxic stimuli. Her research interests include cellular and molecular neuroscience, genetics/genomics, and addiction. Shari is based in the New York office.

Editorial assessment and review synthesis

**Editor's summary and assessment**	The authors recently established platforms for CRISPRi and CRISPRa screens in iPSC-derived neurons (15, 16). This approach has been hard to extend to cultured microglia because it involves lentivirus, which microglia don't take up well. You can introduce the gRNAs at the iPSC stage, but since most microglial differentiation protocols are long, this introduces bottlenecks that can skew the representation of the gRNA library. Here, they developed a different approach for the generation of iPSC-derived microglia by generating a human iPSC line inducibly expressing six transcription factors that enable the generation of microglia-like cells in a rapid and efficient eight-day protocol. Then, they integrated inducible CRISPRi/a machinery into this cell line and screened for effects on survival, phagocytosis, inflammatory activation, and single-cell gene expression.<br><br>Their new protocol involves direct conversion of iPSCs to microglia via expression of six TFs that are highly expressed during microglial development and in adult microglia, but not monocytes and macrophages. They developed a 3-step, 8-day differentiation protocol, and confirmed expression of microglial markers via RNA-seq. They phagocytosed fluorescent beads and rat synaptosomes. They took on an activated morphology when treated with LPS, and had the expected gene expression changes. They secreted the right cytokines.<br><br>They were able to put in the CRISPRa/I machinery and validate that it worked. First they looked at survival. Interestingly, the genes involved in microglial survival were different than those for neurons.<br><br>They looked at modifiers of LPS-induced inflammatory activation using cell surface levels of CD38 as a readout.<br><br>They looked at modifiers of phagocytosis of synaptosomes, and followed up on one, PFN1.<br><br>Then, they selected 38 hit genes of interest, most of which had phenotypes in more than one of the large-scale primary screens for characterization in a CROP-seq screen, which couples CRISPRi perturbation to single-cell RNA sequencing. They found a cluster with disrupted differentiation. They found a

	cluster similar to DAM, for example. And because of the CRISPR, they could identify regulators of the various cell states.<br><br>The new differentiation protocol coupled with the CRISPR screens make this of high interest, and I think the single-cell data are especially exciting.
**Editorial synthesis of reviews**	R1 (microglia) is very positive overall. They should directly compare their differentiation protocol to real human microglia. The comments are all fairly minor and addressable.<br><br>R2 (CRISPR screens) is also positive overall. They need to improve the stats. R2 wants more detail about the differentiation protocol, and how it improves on earlier methods. They should do a timecourse for the effectiveness of the CRISPR system. R2 is critical of their use of a “druggable” genome library, since they don’t follow this up with any drug experiments. Some deeper biological insight would be nice. Overall, R2 has a lot of comments, but they seem addressable.<br><br>R3 (methods for iPSC-derived microglia) is also very positive overall. R3 has some addressable questions about the differentiation approach.

Editorial recommendation

Journal	Recommendation	Comment
Nature	Revision not invited	Following editorial assessment of the paper and reviewer reports it was felt that the conceptual advance is not sufficient for further consideration at Nature.
Nature Neuroscience	Major revisions	The reviews were very positive and addressable overall, and we feel that the paper will be well-received by the community.
Nature Communications	Major revisions with limited extension	Nature Communications would also welcome a revision of the paper, and would require a more limited scope than Nature Neuroscience.

Next steps

Recommendation Summary

- Option 1: Prepare a revision for Nature Neuroscience
- Option 2: Prepare a revision for Nature Communications

Revision

To follow our recommendation, please upload the revised manuscript, along with your point-by-point response to the reviewers' reports and editorial advice **using the link provided in the decision letter**.

Revision checklist

- Cover letter, stating to which journal you are submitting
- Revised manuscript
- Point-by-point response to reviews
- Updated **Reporting Summary** and **Editorial Policy Checklist**
- Supplementary materials (if applicable)

Submission elsewhere

Within the Nature Portfolio

Springer Nature provides authors with the ability to transfer a manuscript within the Nature Portfolio, without the author having to upload the manuscript data again. To use this service, please **follow the transfer link provided in the decision letter**.

Note that any decision to opt in to *In Review* at the original journal is not sent to the receiving journal on transfer. You can opt in to *In Review* at receiving journals that support this service by choosing to modify your manuscript on transfer.

To a journal outside of Nature Portfolio

If you choose to submit your revised manuscript to a journal at another publisher, we can share the reviews with another journal outside of the Nature Portfolio if requested. You will need to request that the receiving journal office contacts us at guidedOA@nature.com. We have included editorial guidance below in the reviewer reports and open research evaluation to aid in revising the manuscript for publication elsewhere.

Annotated reviewer reports

The editors have included some additional comments on specific points raised by the reviewers below, to clarify requirements for publication in the recommended journal(s). However, please note that all points should be addressed in a revision, even if an editor has not specifically commented on them.

Reviewer #1	
**Reviewer #1**	This reviewer has not chosen to waive anonymity. The reviewer's identity can only be shared with representatives of an established journal editorial office.
**Reviewer #1 expertise**<br>Summarised by the editor	microglia
**Editor's comments about this review**	The reviewer provides a positive and addressable report overall.
**Reviewer #1 comments**	
**Overview**	**The study from Dräger et al. describes a novel protocol for the generation of microglia-like cells from human iPSCs (iTF-Microglia), combined with a CRISPR-based screening platform for functional studies of genetic perturbations in vitro. The manuscript is clearly written and well explained. The study provides a remarkable methodological advance, as the protocol described for cell differentiation is considerably shorter than most existing ones, and the integration of the inducible CRISPRi/a machinery overcomes the necessity to transduce mature microglia with lentiviruses, which is notoriously difficult. The authors validate their method by providing comparisons with another established differentiation protocol (Brownjohn et al. 2018). However, although some of the features observed in iTF-Microglia overlap with human microglial phenotypes described in the literature, a direct comparison between iTF-Microglia and human microglia, for example by single-cell transcriptomics, is not provided. Therefore, it remains unclear to what extent iTF-Microglia are similar to microglia in human brains.**<br>**- Out of the six transcription factors shortlisted for the development of the iTF-Microglia tool, the rationale underlying only 5 of them is provided (ll. 136-139),**

while the choice of IRF-5 is not explained.

-

- The authors validated the response of iTF-Microglia to LPS by upregulating CD38 (ll. 289-290). Subsequently, to screen for modifiers of microglia activation, iTF-Microglia were derived, stained for CD38, and FACS-sorted for NGS (l. 296-onwards). In this experiment, did CD38-expressing iTF-Microglia represent "spontaneously activating" cells? Why was the experiment not performed after LPS stimulation?

-

- Related to the above, the authors should mention that CD38 has multiple roles in microglia beyond microglial activation. In fact, CD38 seems to connect activation status to survival, as suggested by Mayo et al. 2008 (ref. 40 in the submitted manuscript).

-

- The authors present their finding that CSF1R knockdown in iTF-Microglia increases phagocytosis while decreasing survival as "unexpected" (l. 334). It is not clear which of these two findings was "unexpected", or if the authors refer to both. In any case, some discussion would be desirable. In Pons et al. 2021 (PMID: 33402196), conditional CSF1R KO does not impair microglial survival or proliferation in mice. Pons et al. suggest that a compensating system might be in place after the KO, involving TREM2/beta-catenin and IL-34. The authors could test if a similar signature is activated in iTF-Microglia, as well as in Brownjohn-iMG.

-

- l. 375. The authors state that they introduced a library of 82 sgRNAs: 2 sgRNAs targeting each selected gene, and 4 non-targeting controls. This does not quite correspond to 38 selected genes + 4 controls (80 in total). Please clarify

Language and style:

- The Introduction reads at times too literary (e.g. ll. 63-64), at times colloquial ("We now know", l. 66), and is somewhat long overall. Consider shortening.

- l. 275: "Oct4" is a mouse gene, and should thus be in Italics.

- l. 296: Fig. 4D – should be lowercase "d".

- l. 332: "mutations in which cause ALS" – "mutations that cause ALS"?

- l. 415: period missing after "CXCL10".

- ll. 471, 472: CCL13 "high" and "low" – should appear as superscripts, instead of subscript.

	**- l. 497: "nontoxic" non-toxic**<br>**- In several places (e.g. ll. 666, 694, Fig. 7), the concentration unit ("nM") is written without space between the number and the unit. There must be a space .**<br>**- l. 818: Remove space between comma and bracket.**<br>**- Extended Data Fig. 1, thousands should be separated by commas.**<br>**- Extended Data Fig. 3c IBA1 on the Y axis should be capitalized; also, in the legend, both IBA-1 and IBA1 (without hyphen) are used. Please check the manuscript for consistency .**<br>**- Extended Data Fig. 7 "labelled" (British English) and "labeled" (American English) both appear. For Nat. Neuroscience, AE is preferred.**

Specific comments

#	Reviewer comment	Editorial comment
1	**a direct comparison between iTF-Microglia and human microglia, for example by single-cell transcriptomics, is not provided. Therefore, it remains unclear to what extent iTF-Microglia are similar to microglia in human brains.**	Nature Neuroscience will require this comparison.

Reviewer #2

**Reviewer #2**	This reviewer has not chosen to waive anonymity. The reviewer's identity can only be shared with representatives of an established journal editorial office.
**Reviewer #2 expertise**<br>Summarised by the editor	CRISPR screens
**Editor's comments about this review**	The reviewer provides an overall constructive and positive report.

Reviewer #2 comments	
Overview	**Overall significance**<br><br>Drager et al introduces a differentiation protocol for the generation of microglia like cells based on the inducible expression of six transcription factors (TFs). They show that these microglia respond to inflammatory stimuli and are capable of phagocytosis. Then, they describe three pooled CRISPR screens to look for genes that control the survival, activation, and phagocytosis of microglia. Finally, they combine a small CRISPRi pooled screens with single-cell RNA sequencing to examine distinct states and genetic regulators.<br><br>**Impact**<br><br>Overall, the work is exciting and the authors' concept of identifying the factors controls different aspects of microglia is intriguing. However, there are a number of concerns over their experimental design and data presentation that need to be addressed in a revised manuscript.<br><br>**Strength of the claims**<br><br>Major comments:<br><br>1. Statistical analysis of most data is missing or incomplete. In certain cases, this leads to data misinterpretation (see #3 below).<br><br>2. The authors presented a novel protocol for generating microglia like cells from iPSCs. However, there is only a short statement in the text saying that "the TFs used in this protocol for inducing microglia differentiation were chosen because that they were highly expressed in adult microglia, but not monocytes and macrophages, based on existing transcriptomic and developmental data". Data analysis supporting this statement is missing and would be helpful to see. Regarding the 6 factors, did the authors examine if a subset of the 6 factors is also sufficient? It would be helpful to also understand how much of each factor is needed. Since the system is dox-inducible, it should be straightforward to test this (with all 6 factors and subsets). Also, the advantage of this new protocol is unclear. Several other protocols are available for generating microglia from iPSCs, such as McQuade et al., 2018 and Speicher et al., 2019. The authors should compare their protocol to other existing ones and explain why they develop and use this new protocol.

3. As shown in Fig 3, the constitutive CRISPRi system has robust knockdown at Day 15 (~95% of non-targeting). The inducible CRISPRi system seems to have 60% knockdown (compare panel c to d). The main text mischaracterizes this as “slightly reduced knockdown”. A 8-fold difference – 5% gene expression vs. 40% gene expression — is in no way “slight”. In general, the main text omits quantitative claims which makes it a bit jarring to see that the data does not really support the text upon careful inspection of the figures. The authors should make an effort to be very critical with their own writing and, when possible, just include the actual numbers.

4. It is inappropriate to make conclusions about different CRISPRi/a systems using just one gene target since different genes can have wide variation (c.f. Yeo et al., Nature Methods, 2018; Figure 4). Please test at least 3 genes with multiple sgRNAs for each system presented.

5. Given that the inducible CRISPRi system seems to be much less effective at Day 15, the authors should perform a timecourse experiment to measure expression every 2-3 days over a 3 week period for all 3 systems.

6. If the inducible system is worse than constitutive CRISPRi, why is it used for the screens in Figure 4, 5 and 6? This needs to be justified given the data shown in Figure 3.

7. For the section titled “Identification of modifiers of microglial survival by CRISPRi screens”, the pooled sgRNA library is first introduced into the iPSCs, and then the CRISPR system is turned on at the same time the differentiation begins. This method is not sufficient to support the conclusions made by the authors on the screen for microglia survival factors since gene inhibition mediated by CRISPRi is likely already occurring before the iPSCs became microglia like cells. This is further supported by the observation that around threefold increase in cell number was present at day 4 post-differentiation (Fig 4c). The observed continuous increase in cell number during the first few days suggests that the cells have not differentiated into microglia like cells, suggesting the impact of silencing the targeted genes occurs at a pre-microglia stage. Given the stated purpose of the screen (and the choice of inducible system), the authors should design the experiment to turn on CRISPRi at the later stage of differentiation to achieve the desired purpose (survival factors).

8. In the screen for identifying modifiers of microglial activation, the authors used surface levels of CD38 as a readout for microglial activation after LPS treatment.

What's the criteria for setting the gate to collect CD38 high and low populations (Fig 4d)? The authors should add the FACS plots to show the surface levels of wild type cells before and after activation.

9. Fig 4e and Fig 4f illustrated that knockdown of CDK12 increased CD38 surface levels, suggesting an inhibitory role of CDK12 on inflammation. However, the authors cited a paper (reference 42) to support CDK12's role in promoting inflammation, which is confusing.

10. To identify the modifiers of synaptosome phagocytosis by microglia, the authors conducted parallel CRISPRi and CRISPRa screens with the same sgRNA libraries and found little overlap between CRISPRi and CRISPRa hits. Then, the authors state that this data confirmed their previous finding that overexpression and knockdown screens can provide complementary insights.

The authors cited a paper (reference 49) to support this claim, however, the CRISPRi and CRISPRa screens in reference 49 were designed differently, and the data from that reference does not support the claim. The authors should add more analysis and discussion to explain.

11. The authors used a “druggable genome” library, presumably to find new drug targets. Given that none of the screen hits are followed up using drugs/small molecules, it would be preferable to use a genome-wide library (or a library based on microglia gene expression). Only targeting 10% of the genome (2,000 vs 20,000 genes) provides false confidence that the screen hits are the most relevant to the biological questions proposed. This is a major drawback of the study and all 3 screens.

12. No figures are shown for single-cell/CROP-seq quality control. It is essential to show data regarding read depth, cell capture, singlets, etc. in order to evaluate the quality of the single-cell experiments.

13. Given that NTC cells are found in every UMAP cluster, the authors should quantify the specificity of each perturbation. For each perturbation, how many clusters is it found in? Are any perturbations found only in 1 or 2 clusters? The data presentation (e.g. Figure 7b, which only shows change) makes it difficult to understand the specificity of the perturbations and whether they resulted in a specific microglia state. This is shown for a few perturbations in a non-quantitative manner (Extended Data Figure 5b) but should be quantified and discussed. If the states/perturbations do not cluster, is this expected? Or not?

14. Although I appreciate the work that went into the various screens, it would be great to get some deeper biology and insight into the top screen hits for any of the 3 screens. For example, in the SPP1+ cluster, what are the kinase targets of MAPK14? Does MAPK14 directly control SPP1 expression or (if relevant) its phosphorylation? By what mechanism does CDK12 knockdown enrich CCL13+ cells? If the SPP1+ cluster is “disease relevant”, can the authors show what is the impact of these perturbation on disease phenotypes instead of just changes in UMAP cluster distribution? As written, the interpretation of the CROP-seq screen seems like a list of findings without clear direction.

Minor comments:

1. The "druggable genome" citation, reference #32, is incorrect.

2. Nomenclature for induced neurons and microglia should be consistent. If using “iNeurons” for neurons derived with the Neurogenin transcription factor, then perhaps “iMicroglia” would be better than “iTF-Microglia”.

Specific comments

#	Reviewer comment	Editorial comment
1	Statistical analysis of most data is missing or incomplete.	Nature Neuroscience and Nature Communications will both require this to be fully addressed.
2	The authors should compare their protocol to other existing ones and explain why they develop and use this new protocol	Nature Neuroscience and Nature Communications will both require this comparison.
3	Please test at least 3 genes with multiple sgRNAs for each system presented.	Nature Neuroscience would like for this to be addressed.
4	the authors should perform a timecourse experiment to measure expression every 2-3 days over a 3 week period for all 3 systems.	Nature Neuroscience would like for this to be addressed.

5	**The authors used a "druggable genome" library, presumably to find new drug targets. Given that none of the screen hits are followed up using drugs/small molecules, it would be preferable to use a genome-wide library (or a library based on microglia gene expression).**	Nature Neuroscience expect the authors to respond to this comment, but not necessarily to repeat everything using a different library.<br>Nature Communications does not require the authors to use a different library.
6	**Although I appreciate the work that went into the various screens, it would be great to get some deeper biology and insight into the top screen hits for any of the 3 screens.**	Nature Neuroscience would like to see some of this "deeper biology" added. The authors can choose the direction for these experiments.

Reviewer #3

**Reviewer #3**	This reviewer has not chosen to waive anonymity. The reviewer's identity can only be shared with representatives of an established journal editorial office.
**Reviewer #3 expertise**<br>Summarised by the editor	Deriving microglia from iPSCs
**Editor's comments about this review**	The reviewer has provided a constructive and supportive report.

Reviewer #3 comments

**Overview**	**Overall significance**<br>**In this manuscript, Dräger and colleagues develop a new rapid transcription-factor based method to generate microglia-like cells from iPSCs in order to perform CRISPRa and CRISPRi screens to identify genes that modify microglial survival, activation, and phagocytosis. This is an important, timely, and carefully executed study that will provide the field with a powerful new way to manipulate and study human microglia. In addition, the authors have used their expertise in CRISPR screening to identify several genes that influence microglial function and have further validated some of these hits using a secondary non-TF based microglial**

differentiation protocol. For the most part the figures and data presented are very clear and compelling and the conclusions appropriate. There are however a couple additional analyses that if added would further broaden the implications of this manuscript and provide the field with a more complete understanding of the utility of the TF-based differentiation approach, specifically:

1) It appears from the methods and Fig 1b that doxycycline is maintained in the media throughout the differentiation and experimentation. It is important to also clarify this within the results section and to include a discussion of this caveat. What happens if doxycycline is removed from the media after differentiation? Is the transcriptome and function of the iTF-microglia maintained or do they revert to a less microglial-like state? As doxycycline can itself affect microglial activation it would be helpful to know if this can be removed from the media prior to experimentation. It is not necessary to repeat the screens in the absence of Dox but some additional analysis of the dependence on Dox for maintaining the iTF-microglial phenotype would be extremely helpful.

2) In Figure 1e, the authors compare the transcriptome of d9 and d15 iTF-microglia to iPSCs. This provides a helpful contrast and suggests there is good upregulation of many microglial genes. However, it does not provide information about how similar iTF-microglia are to prior ontogeny-based protocols or cultured brain-derived human microglia. Additional comparisons to RNA seq datasets from a couple previously established iMGL protocols (Brownjohn plus one other) and human cultured microglia (Gosselin et. al., Science) would be extremely helpful to include in Figure 1. PCAs of total gene expression for these comparisons that both include and exclude the iPSC contrast would also be helpful (in supplemental would be fine for these). Clearly iTF-microglia are microglia-like and offer a very important advantage for CRISPR screens but understanding if there are also any transcriptional deficiencies or advantages in comparison to longer protocols would be highly informative. The authors do include validation of some CRISPR phagocytosis hits using the Brownjohn method and a supplementary figure comparing the Brownjohn dataset +/- LPS which is very helpful. However, inclusion of a baseline (unstimulated) comparison to other datasets in the heatmap in Figure 1e and a PCA would greatly improve the readers' understanding of this approach. Likely there will be important differences that need to be acknowledged and discussed, some of which may be due to doxycycline (see above comment), others may be due to the inclusion of TGFb,which is not included in the Brownjohn protocol or Gosselin dataset, and others will be due to the TF-based methods. All iMGL protocols have their pros and cons and understanding how this new TF-based approach fits into the growing set of protocols will be highly informative for the field.

Minor Points:

Figure 2A, the starting density and morphology of the DMSO vs CytoD cultures looks quite different. Where these treatments performed in parallel? If so, is the morphology and density variable from well to well of the same differentiation?

The authors first test the functionality of CRISPRa by inducing CXCR4. Is there a specific reason they chose to test this specific receptor?

The authors findings that TGFbr2 knockdown reduces IBA and disrupts microglial differentiation makes sense in light of evidence that TGFb is important for murine microglial development. I'd suggest adding that point and relevant references to the this part of the results.

Impact

Yes, this manuscript will likely impact thinking in this area. In particular the development and validation of a shortened TF-based microglial differentiation approach will likely be widely applied for additional subsequent screens.

Strength of the claims

Yes, although see comments above regarding the need for some additional bioinformatic comparisons to existing microglial differentiation methods.

Reproducibility

The quality and statistical analysis of the data are strong.

Specific comments

#	Reviewer comment	Editorial comment
1	What happens if doxycycline is removed from the media after differentiation?	Nature Neuroscience would like the authors to address this.
2	does not provide information about how similar iTF-microglia are to prior ontogeny-based protocols or cultured brain-derived human microglia. Additional comparisons to RNA seq datasets from a couple previously established iMGL protocols (Brownjohn plus	Both Nature Neuroscience and Nature Communications would require the comparison of the transcriptional profiles of iTF-microglia to human microglia

	**one other) and human cultured microglia (Gosselin et. al., Science) would be extremely helpful to include**	cultured with other protocols.

Open research evaluation

Data availability
**Data availability statement**
Thank you for including a Data Availability statement. We noticed that not all datasets reported in the paper are included in this statement. The data availability statement must make the conditions of access to the "minimum dataset" that are necessary to interpret, verify and extend the research in the article, transparent to readers. More information about our data availability policy can be found here: https://www.nature.com/nature-portfolio/editorial-policies/reporting-standards#availability-of-data<br><br>See here for more information about formatting your Data Availability Statement: http://www.springernature.com/gp/authors/research-data-policy/data-availability-statements/12330880
**Mandatory data deposition**
For your RNA sequencing data, submission to a community-endorsed, public repository is mandatory for publication in a Nature Portfolio journal and is best practice for publication in any venue. Accession numbers must be provided in the paper. Examples of appropriate public repositories are listed below:<br><br>-Gene Expression Omnibus (Microarray or RNA sequencing data)<br>-Sequence Read Archive (high-throughput sequence data)<br>-The European Nucleotide Archive (ENA)<br><br>More information on mandatory data deposition policies at the Nature Portfolio can be found at http://www.nature.com/authors/policies/availability.html#data<br><br>Please visit https://www.springernature.com/gp/authors/research-data-policy/repositories/12327124 for a list of approved repositories for each mandatory data type.
Ethics

Manuscripts that report experiments involving the use of human embryos and gametes, human embryonic stem cells and related materials, and clinical applications of stem cells must include confirmation that all experiments were performed in accordance with relevant guidelines and regulations.

Please ensure your manuscript includes an ethics statement identifying the institutional and/or licensing committees approving the experiments and describing any relevant details. The ethics statement must also confirm that informed consent was obtained from all recipients and/or donors of cells or tissues, where necessary, and describe the conditions of donation of materials for research, such as human embryos or gametes.

Reporting & reproducibility

We encourage you to share your step-by-step experimental protocols on a protocol sharing platform of their choice. The Nature Portfolio's Protocol Exchange is a free-to-use and open resource for protocols; protocols deposited in Protocol Exchange are citable and can be linked from the published article. More details can be found at www.nature.com/protocolexchange/about

Statistics and data presentation

To improve reproducibility of your analyses, please provide details regarding your treatment of outliers.
